# MicroRNAs in brain development and function: a matter of flexibility and stability

**DOI:** 10.3389/fnmol.2014.00005

**Published:** 2014-02-07

**Authors:** Philipp Follert, Harold Cremer, Christophe Béclin

**Affiliations:** Institut de Biologie du Développement de Marseille, Aix-Marseille Université – Centre National de la Recherche ScientifiqueMarseille, France

**Keywords:** microRNA, neurogenesis, neural stem cells, fate determination, synaptogenesis, synaptic function, LTP

## Abstract

Fine-tuning of gene expression is a fundamental requirement for development and function of cells and organs. This requirement is particularly obvious in the nervous system where originally common stem cell populations generate thousands of different neuronal and glial cell types in a temporally and quantitatively perfectly orchestrated manner. Moreover, after their generation, young neurons have to connect with pre-determined target neurons through the establishment of functional synapses, either in their immediate environment or at distance. Lastly, brain function depends not only on static circuitries, but on plastic changes at the synaptic level allowing both, learning and memory. It appears evident that these processes necessitate flexibility and stability at the same time. These two contrasting features can only be achieved by complex molecular networks, superposed levels of control and tight interactions between regulatory mechanisms. Interactions between microRNAs and their target mRNAs fulfill these requirements. Here we review recent literature dealing with the involvement of microRNAs in multiple aspects of brain development and connectivity.

## INTRODUCTION: MICRORNA GENESIS AND FUNCTION

MicroRNAs are small RNA molecules of around 22 nucleotides, processed from longer primary transcripts (pri-miRNAs) in successive maturation steps. MicroRNA genes contain an imperfect palindromic sequence that creates a secondary stem–loop structure within the pri-miRNA. This stem–loop structure contains the mature microRNA and its passenger strand (Denli et al., 2004; Gregory et al., 2004; Landthaler et al., 2004; Han et al., 2006) and serves as substrate for two double-strand RNases, Dicer and Drosha (Carmell and Hannon, 2004). Targeting occurs by partial complementarity between the mRNA’s 3’UTR and a 6–8 nucleotides long sequence at the 5’ end of the microRNA. This partial complementarity allows a single microRNA to target multiple mRNAs simultaneously and, vice versa, a single mRNA may be regulated by different microRNAs (Klein et al., 2005; Kosik, 2006). Thus, bioinformatic predictions and proteomic evidence indicate a vast amount of potential microRNA/mRNA interactions (Bartel, 2009). In addition to other regulatory mechanisms (feedback loops among transcription factors, epigenetic mechanisms, etc), microRNAs have been implicated in the control of neurogenesis and brain function. We will discuss several examples in this review.

## MicroRNAs CONTROLLING NEUROGENESIS: FROM STEM CELLS TO NEURONS

Maintenance and differentiation of neural stem cells is controlled by the equilibrium between the relative amounts of key proteins that promote or inhibit entry into the neurogenic program. Multiple examples show that this equilibrium is achieved, at least in part, by microRNAs that act in complex feedback loops with their targets.

### THE TLX SYSTEM: STABILIZATION BY FEEDBACK LOOPS

**FIGURE 1:**
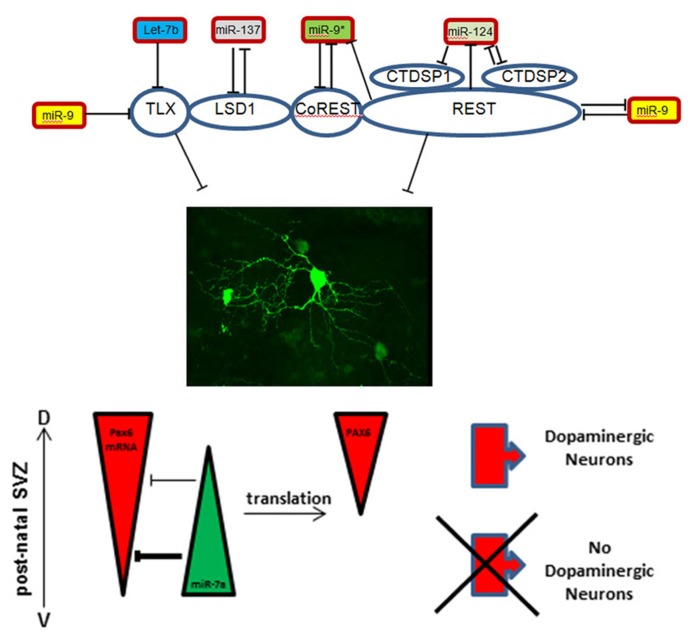
**Schematic representation of microRNA-target interactions in the control of maintenance versus differentiation in the neural stem cell pool**.

One example for such complex regulation is provided by control and interactions of the orphan nuclear receptor Tailless (TLX). TLX is expressed in stem cells of the developing and adult brain where it controls their maintenance and proliferation (Shi et al., 2004; Liu et al., 2008; Zhang et al., 2008). MiR-9 is a highly brain enriched microRNA that targets and regulates TLX (Zhao et al., 2009) expression and is itself negatively regulated by the nuclear receptor (Zhao et al., 2009; **Figure 1**). Moreover, two members of the let-7 microRNA-family also control TLX expression, thus acting upstream of the TLX/miR-9 feedback loop (Zhao et al., 2010, 2013). Interestingly, both miR-9 and let-7b also share CyclinD1, another key cell cycle regulator during neurogenesis, as a target (Guo et al., 2013; Zheng et al., 2013). Finally, during cortical development TLX acts in concert with the lysine specific de-methylase 1 (LSD1) that is controlled by miR-137, which, in turn, is repressed by TLX dependent recruitment of LSD1 to the microRNA locus (Sun et al., 2011; **Figure 1**).

### REST INHIBITION TO OPEN THE DIFFERENTIATION LOCK

A second example for the sophisticated regulatory interactions that control neural stem cells status implicates the zinc finger protein REST (RE1-silencing transcription factor). REST and its co-repressor CoREST are part of a protein complex that binds to the so-called RE1 site of target promoters and thereby down-regulates neuronal genes in non-neural tissues (Andres et al., 1999; Ballas and Mandel, 2005; Bithell, 2011). The REST complex contains additional proteins like the phosphatases CtdspL, Ctdsp1, Ctdsp2 (Yeo et al., 2005) and, interestingly, LSD1, providing an intersection with the TLX system (Lee et al., 2005). Since the REST complex opposes neuronal differentiation, and thus maintains the immature state (Ballas and Mandel, 2005), it has to be released from its binding site to allow neurogenesis. As for TLX, miR-9 targets and down-regulates REST while its counterstrand miR-9* targets CoREST (Packer et al., 2008; **Figure 1**). Conversely, the miR-9/miR-9* genomic loci both contain RE1 sites upstream of the protein coding sequence and are regulated by the REST complex (Packer et al., 2008).

Another regulator of the REST control system is miR-124, one of the most abundant microRNAs in the brain. During development miR-124 promotes neuronal differentiation by targeting REST, again implicating a feedback loop since REST itself acts as inhibitor of miR-124 expression (Conaco et al., 2006; Visvanathan et al., 2007). In addition, a synergistic function of miR-124 and miR-9*, the passenger strand of miR-9, has been reported (Yoo et al., 2009). Both microRNAs repress the subunit BAF53a of the neural-progenitor-specific BAF (npBAF) chromatin-remodeling complex, which allows a switch to the BAF53b subunit (Yoo et al., 2009). This subunit switch is important for post-mitotic phases of neural development. Additionally, miR-124 was shown to target the RNA-binding protein Ptbp1, a repressor of neuron-specific splicing (Makeyev et al., 2007) as well as laminin γ1 and integrin β1, both repressed during neuronal differentiation (Cao et al., 2007). Finally, miR-124 was shown to be involved in postnatal neurogenesis through its inhibition of the neural stem cell (NSC) maintenance factor Sox9 (Cheng et al., 2009). Taken together this indicates that miR-124 promotes neuronal differentiation, both, during embryonic development and in postnatal stages, thereby acting on multiple molecular layers from transcription and splice factors to extracellular matrix molecules.

### EPIGENETIC MECHANISMS

Surprisingly, in contrast to its above-described pro-neurogenic role in the embryo, miR-137 has been implicated in the maintenance of stem cell proliferation in the adult forebrain through cross-talk with epigenetic mechanisms involving MeCP2 and Ezh2 (Szulwach et al., 2010).

Moreover, miR-184 is another microRNA which links epigenetic processes to neurogenesis (Liu et al., 2010). The authors reported that the loss of methyl binding protein MBD1 increased the expression of miR-184 and identified Numblike (Numbl), a Notch1 antagonist important for survival of SVZ derived neuroblasts (Kuo et al., 2006), as a direct target.

### DETERMINATION OF NEURONAL FATE

A key feature of brain development is that common neural stem cells are able to generate a large diversity of cell types. The role of microRNAs on lineage and subtype specification in the brain just starts being explored. During postnatal neurogenesis miR-7a has been reported as an important contributor to fate specification of OB dopaminergic inter-neurons. The regulation by miR-7a impacts on gene dosage and the precise expression pattern of the transcription factor Pax6 which is a critical dopaminergic fate determinant in the SVZ (Hack et al., 2005; de Chevigny et al., 2012b). This is part of the control system determining neurotransmitter phenotype of OB inter-neurons (de Chevigny et al., 2012a). Interestingly, during cortex development miR-7a was found to promote oligodendrocyte generation by targeting Pax6 and NeuroD4 (Zhao et al., 2012). Thus, mir-7a is able to control different types of fate decision by controlling the same targets in different transcriptional contexts (**Figure 1**).

Mir-133 has been implicated in midbrain dopaminergic differentiation *in vitro* through regulation of Pitx3. Moreover, Parkinson’s patients have been shown to be deficient for this microRNA, suggesting a feedback circuit in the fine-tuning of dopaminergic behaviors (Kim et al., 2007). However, these findings have been challenged by the recent observation that miR-133b-deficient mice show normal numbers and function of dopaminergic neurons (Heyer et al., 2012). Thus, the situation needs clarification.

Another interesting microRNA in regard to specification events is miR-34a. This microRNA is reported to promote generation of post-mitotic neurons from isolated mouse embryonic NSCs by targeting the NAD-dependent deacetylase sirtuin-1 (Sirt1; Aranha et al., 2011). In contrast, miR-34a is reported to enhance Notch1 signaling in neural progenitors, by repressing the Notch pathway repressor Numbl that ultimately antagonizes neuronal differentiation (Fineberg et al., 2012). Taken together, this might indicate that miR-34a acts strongly context dependent based on the transcriptional and cellular environment.

In conclusion, investigation of regulatory interactions between microRNAs and their targets in the control of neurogenesis revealed complex regulatory circuits based on feedback regulations, synergistic actions of several microRNAs and intersections between signaling systems.

## MicroRNAs AT THE SYNAPSE

Synapses are the main structures that allow communication between neurons. Synapses of a given neuron may coexist in different states, differing in strength, thus the capacity of the synapse to respond to presynaptic release of neurotransmitter. The property of a synapse to modify its strength is called synaptic plasticity which comes in two flavors. Long term potentiation (LTP) is induced by high frequency stimulation of presynaptic neurons (Bliss and Lomo, 1973) and results in an increase in the density of AMPA receptors at the post-synaptic membrane, leading to enhanced Na^+^ flux (Johnston et al., 2003; Malenka and Bear, 2004). This, in turn, increases the likelihood of synaptic signal transmission. LTP is specific to a given synapse and spreading to the neighboring synapses is efficiently inhibited. In contrast, during long term depression (LTD), low-frequency stimulation decreases the strength of a synapse (Massey and Bashir, 2007). Overlying these processes, homeostatic mechanisms exist at the pre- and post-synaptic compartments that dampen these opposing phenomena (LTP and LTD) to avoid hyper or hypo-excitability of synapses in response to permanent high or low-frequency stimulation (Malenka and Bear, 2004; Lee et al., 2010). This situation, where synapses exhibit variable strength in the brain, draws a landscape of favored neuronal circuits where transmission will occur with higher probability than others.

Establishment of defined neuronal circuits in particular states is considered to be the basis of both, memory and learning. For long-term memory, information has to be stably stored over prolonged periods, implying a high degree of stability of a given circuit state. In contrast, learning in response to stimuli from the outside world has to be associated with rapid changes at the synaptic level leading to rapid changes in circuit status. It is evident that these seemingly opposing cellular processes occur also at the molecular level. Thus, regulatory fine-tuning mechanisms must exist, that allow synaptic stability and flexibility at the same time.

### MicroRNAs REGULATING FORMATION AND STABILITY OF THE SYNAPSE

Molecularly, LTP (the situation is not clear for LTD) is characterized by a change in the biochemical composition of the activated synapse, with specific recruitment of key synaptic proteins. These mechanisms are mainly under the control of CamKII signaling. It has recently been shown that the synaptic accumulation of several important LTP-inducing proteins is a consequence of local synaptic translation (Hornberg and Holt, 2013; Swanger et al., 2013) establishing a link between the protein content of a given synapse and its strength.

As for regulation at the stem cell level, over the past years a variety of mRNA/microRNA interactions have been described, that fulfill the requirement of providing flexibility and stability at the same time. Indeed, a subset of microRNAs was found strongly enriched in synapse preparations of forebrain tissue (Lugli et al., 2008). Moreover, MOV10, a helicase that is part of the RISC complex (Chendrimada et al., 2007), is accumulated at synapses and actively degraded upon activity. Absence of MOV10 displaced a subset of major synaptic mRNA into the polysomal fraction, demonstrating microRNA-mediated control of translation at the synapse (Banerjee et al., 2009).

In parallel to these more global approaches, several specific microRNAs were shown to be involved in synaptic plasticity, whereby they act at different levels. In some cases they participate in silencing synapses by inhibiting expression of structural proteins while in other cases they favor synaptic potentiation. Moreover, some microRNAs have been involved in synaptic homeostasis, by limiting the over-expression of synaptic proteins upon activation. Interestingly, several microRNAs that control synaptic protein expression have been implicated in drug addiction.

**FIGURE 2:**
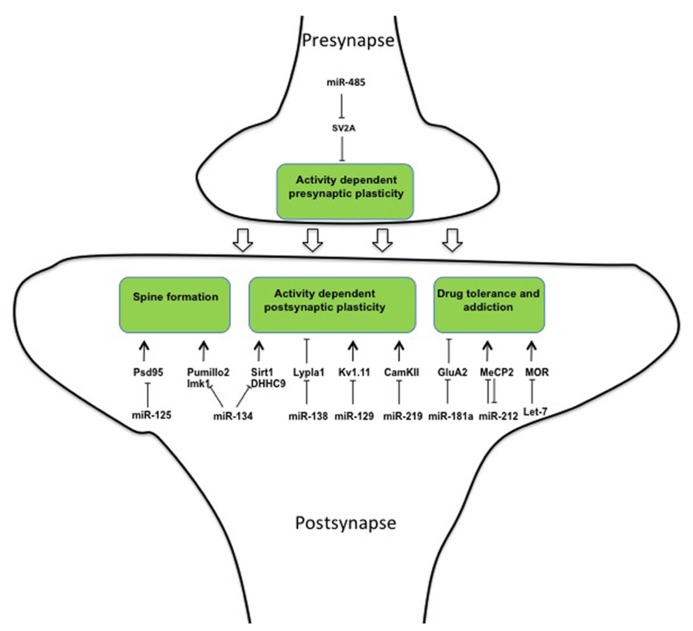
**MicroRNAs regulate different aspects of synapse formation, stabilization, and plasticity**.

Several microRNAs prevent expression of synaptic proteins in the presence of the corresponding mRNAs. Upon stimulation relieve of this translational block allows the rapid activation of the synapse. The first microRNA shown to be involved in synapse formation was miR-134 (Schratt et al., 2006). Its precursor is transported specifically to dendrites via binding to the DEAH-box helicase DHX36 (Bicker et al., 2013). Once arrived in the dendrites, pre-miR-134 is processed into mature miR-134, which inhibits spine formation in cultured hippocampal neurons (Schratt et al., 2006) and dendritogenesis in cortical neurons (Christensen et al., 2010) via the kinase Limk1 and the translational repressor Pumillo2 (Schratt et al., 2006; Fiore et al., 2009). Upon neuronal activation, the inhibitory effect of miR-134 is relieved and spine formation occurs (Schratt et al., 2006). In line with its role in opposing spine formation, miR-134 was recently shown able to impair synaptic plasticity through the inhibition of SIRT1 gene in a gain-of-function setting (Gao et al., 2010; **Figure 2**).

However, two recent papers interrogate the assumption that miR-134 is a general opponent to active excitatory synapses formation. First, it was shown that inhibition of miR-134 reduces spine density in hippocampal pyramidal neurons *in vivo* (Jimenez-Mateos et al., 2012) thereby protecting from epileptic seizure (Jimenez-Mateos et al., 2012). This suggests a pro-synaptogenic role of the microRNA in excitatory neurons. Second, whereas all these previous miR-134 related observations were made in excitatory neurons, a recent paper showed that activity of miR134 in cortex is restricted to inhibitory GABAergic inter-neurons where it down-regulates DHHC9, the palmitoyltransferase of the regulatory GTPase HRAS (Chai et al., 2013). To reconcile these contrasting results the authors propose that miR-134 exerts its function on excitatory neurons indirectly, through the associated inter-neurons (Chai et al., 2013).

### MicroRNAs REGULATING SYNAPTIC PLASTICITY

Palmitoylation is a post-translational modification that is commonly used to mediate activity-dependent changes in synapses (Kang et al., 2008). MiR-138 is present at the post-synapse where it regulates dendritic spine morphology through translational inhibition of the de-palmitoylating enzyme Lypla1 (Siegel et al., 2009). Moreover, miR-125 was found to regulate synaptic plasticity in cortical neurons through translational inhibition of the post-synaptic protein PSD-95. Interestingly, binding of miR-125 to PSD-95 is mediated by the phosphorylated form of FMRP, the gene responsible for the fragile-X syndrome (Muddashetty et al., 2011). In response to stimulation of metabotropic mGluR receptors FMRP is dephosphorylated and miR-125 is released from PSD-95 3’UTR mRNA, which can then be translated (**Figure 2**).

Kv1.1 is a voltage-gated potassium transporter that controls action potential frequency (Brew et al., 2003). Exact dosage of this transporter is important as even a mono-allelic mutation induces episodic ataxia in human patients (Zerr et al., 1998) and appropriate levels of Kv1.1 protein at synapse are assured by positive and negative regulation of its translation. In this system the neuron-specific microRNA miR-129 binds and inhibits Kv1.1 mRNA translation (Sosanya et al., 2013). However, miR-129 competes for Kv1.1 mRNA-binding with the RNA-binding protein HuD, which acts as a positive regulator of Kv1.1 protein expression. The master regulator of this system, which orchestrates between positive and negative regulation, is the mTOR kinase. Activity of mTOR results in increased amounts of intra-cellular HuD that displaces miR-129 from Kv1.1 mRNA, thus allowing translation to occur (Raab-Graham et al., 2006; **Figure 2**).

MicroRNA miR-219 expression in the prefrontal cortex parallels expression of the NMDA-receptor. Moreover, CamKII, a major mediator of LTP and NMDA signaling, was shown to be a direct target of miR-219 (Kocerha et al., 2009). Finally, miR-219 down-regulation alleviates behavioral modifications associated with alterations in NMDA-receptor signaling, in accordance with a functional role of miR-219 in synaptic plasticity (Kocerha et al., 2009). Thus, a multitude of regulatory interactions between microRNAs and target genes have been implicated in the negative control of synapse formation and transmission.

However, there is also evidence that microRNAs promote synaptic plasticity upon activation. Transgenic mice over-expressing miR-132 in forebrain neurons exhibit increased spine density (Hansen et al., 2010) while miR-132 inhibition reduces spine formation (Magill et al., 2010). These results, together with the observation that miR-132 accumulates in response to activity (Nudelman et al., 2010), suggest a positive role for this microRNA for synapse formation and plasticity. However, the situation might be more complicated, since miR-132 has also been shown to inhibit the CpG-binding protein MeCP2 (Klein et al., 2007), an inducer of spine formation.

The inhibitory activity of microRNAs may also be used to dampen structural changes at synapses upon activation and thus be involved in homeostatic plasticity. After stimulation of cultured hippocampal neurons, miR-485 expression was increased at pre-synapses (Cohen et al., 2011). Here, the microRNA was shown to regulate the pre-synaptic protein SV2A (**Figure 2**) and by this to reduce the probability of neurotransmitter release as shown by a lower miniature excitatory synaptic current (mEPSC) frequency. This inhibition in pre-synaptic function partially prevented clustering of post-synaptic proteins such as PSD95 and AMPA receptor subunits (Cohen et al., 2011).

### FROM SYNAPTIC FUNCTION TO DRUG ADDICTION

Several reports demonstrate the involvement of the microRNA pathway in homeostatic plasticity occurring in response to drug intake. Indeed, psychotropic drugs act generally through stimulation of specific synaptic receptors. Repeated stimulation of these receptors reinforces the strength of the involved neuronal circuitries. This leads to compulsive consumption of the drug if the potentiation at the synapse is not dampened. Several microRNAs were shown to be involved in the response to chronic drug exposure and to drug addiction. MicroRNA miR-181a is specifically accumulated at post-synapses of nucleus accumbens. Moreover, its concentration increases during cocaine abuse (Saba et al., 2012). At the post-synapse, one of the miR-181a targets is the AMPA subunit GluA2 (Saba et al., 2012; **Figure 2**). It is known that drug of abuse favors the exchange from GluA2 containing AMPARs to GluA2 lacking AMPARs and this molecular modification at the synapse is required for drug-craving after prolonged cocaine withdrawal (Conrad et al., 2008). It appears possible that this mechanism is responsible for the role of miR-181a in the alterations in “cocaine place preference” (CCP) that have been shown in rodents (Chandrasekar and Dreyer, 2011) and also in the altered neuro-adaptation associated with cocaine abuse (Saba et al., 2012).

Neuro-adaptation leads to profound structural alterations that can, depending on the individual, lead to variations in sensitivity to a drug over time (Bowers et al., 2010; Dacher and Nugent, 2011). This variation explains why some subjects will become addicts and others will not. miR-212 was shown to play a central role in neuro-adaptation and to oppose loss of control toward drug consumption. Upon chronic cocaine exposure miR-212 and its cluster neighbor miR-132 are over-expressed in the dorsal striatum (Hollander et al., 2010). Under extended access to cocaine gain- and loss-of-function experiments showed that miR-212 interfered with the self-administered dose. These results suggest that miR-212 is involved in the dampening of plasticity induced by chronic cocaine exposure, which causes the compulsive behavior. At the molecular level, the action of miR-212 is mediated through the inhibition of a so far unidentified repressor of Raf1, which is itself an activator of CREB. This indirect activation of CREB, reduces the motivational properties of the drug by dampening the reward circuitry (Dinieri et al., 2009). Moreover, miR-212 has been shown to target MePC2, as already mentioned a DNA-binding protein involved in synaptic structural plasticity, providing a parallel pathway accounting for the anti-addictive role of the microRNA toward cocaine (Im et al., 2010). Interestingly, MeCP2 inhibits expression of miR-212 (**Figure 2**), and by this limits the action of miR-212 in the control of cocaine intake, highlighting again the importance of feedback loops in the regulatory actions of microRNAs (Im et al., 2010).

In addition to this considerable amount of information implicating microRNAs in the control of addiction to cocaine, microRNAs are involved in the behavior toward Opioids. These are potent analgesics of considerable clinical value, but have several drawbacks limiting their use, including tolerance and addiction. Opioid signaling is mediated in neurons through the mu opioid receptor (MOR) and tolerance occurs through the decrease in MOR expression at the synapse. He et al. (2010) showed that the microRNA let-7, on one hand, inhibits MOR translation and, on the other hand, accumulates upon chronic morphine treatment in mice (**Figure 2**). Moreover, knocking-down let-7 reduced -but did not entirely prevent- opioid tolerance in treated mice, demonstrating a role of the microRNA in dampening opioid signaling upon chronic stimulation (He et al., 2010).

## CONCLUSION

MicroRNAs have been shown to be implicated in virtually all biological functions ranging from embryonic development, aging, infections, genetic disease to cancer (Tang et al., 2007).

However, microRNAs do in general not have simple functions as on/off switches, but serve whenever fine-tuning of gene expression in space, time and dose is necessary. In the brain the necessity for such fine-tuning is evident (Schratt, 2009). In the stem cell compartment the generation of neurons from initially quite homogeneous stem cells population has to be orchestrated in space and time to generate the thousands of different neuronal and glial cell types in the correct place and number. For proper function, these cells have to form complex cellular circuitries that are tightly regulated at the levels of connectivity and synaptic signal intensity. Here we reviewed the functions of gene and microRNA interactions in different aspects of these processes. We find that many of the microRNAs in the brain are implicated in many aspects of the neurogenic process, thereby regulating different targets sequentially and often synergistically with other microRNAs. Another common feature of these interactions is that they control homeostasis of otherwise fragile systems, thereby often implicating complex feedback loops. Finally, the brain has to react instantaneously to outside stimuli and microRNA mediated control of gene expression allows bypassing the transcriptional control level. Given all these properties and requirements, it is predictable that in the future a multitude of further interactions, loops and functions implicating microRNAs will be described.

## Conflict of Interest Statement

The authors declare that the research was conducted in the absence of any commercial or financial relationships that could be construed as a potential conflict of interest.
